# Feature extraction and machine learning techniques for identifying historic urban environmental hazards: New methods to locate lost fossil fuel infrastructure in US cities

**DOI:** 10.1371/journal.pone.0255507

**Published:** 2021-08-04

**Authors:** Jonathan Tollefson, Scott Frickel, Maria I. Restrepo

**Affiliations:** 1 Department of Sociology, Brown University, Providence, Rhode Island, United States of America; 2 Center for Computation and Visualization, Brown University, Providence, Rhode Island, United States of America; National University of Sciences and Technology (NUST), PAKISTAN

## Abstract

U.S. cities contain unknown numbers of undocumented “manufactured gas” sites, legacies of an industry that dominated energy production during the late-19th and early-20th centuries. While many of these unidentified sites likely contain significant levels of highly toxic and biologically persistent contamination, locating them remains a significant challenge. We propose a new method to identify manufactured gas production, storage, and distribution infrastructure in bulk by applying feature extraction and machine learning techniques to digitized historic Sanborn fire insurance maps. Our approach, which relies on a two-part neural network to classify candidate map regions, increases the rate of site identification 20-fold compared to unaided visual coding.

## Introduction

From the mid-19th to the mid-20th century, many city dwellers in the United States and much of Canada, Europe and Australia relied on gas manufactured from burning coal for street lighting and to light and heat homes and businesses [1]. Most manufactured gas, also referred to as “coal gas” or “town gas,” was produced locally and delivered to consumers through networks of underground pipes. Along these networks, substations housed intermediary gasholders or “gasometers”–large cylindrical structures that stored pressurized gas for distribution to more distant neighborhoods. Populous cities might include several dozen gas production and distribution sites, and by 1900 even smaller, remote cities would have had at least one gas plant [2, 3]. Smaller plants produced gas for individual institutions, including hospitals, colleges, factories, railroad yards, and private estates [3].

United States manufactured gas production peaked in the 1920s [4]. Soon after, as long-distance pipeline technologies increased competition from natural gas fields in California and the American southwest [5], the industry fell into steady decline. By 1950, many of these sites were abandoned; while some adapted for oil and natural gas, others fell into disrepair or were demolished [1]. Today, nearly all former manufactured gas production and storage sites have disappeared, the land they once occupied redeveloped for other industrial, commercial, residential, or public uses. In many cases, redevelopment occurred decades prior to the establishment of federal and state regulations requiring rigorous site investigation, leaving earlier hazardous industrial land uses undiscovered and legacy contamination poorly documented, in many cases hidden beneath multiple iterations of urban land use and reuse [6, 7]. The US Environmental Protection Agency (EPA) puts the total number of gas production plants at between 3,000 and 5,000 nationally [8]; estimates increase ten-fold when accounting for gas distribution sites and small, institutional MGPs [3]. Remediation experts are confident that only a small fraction of the once-expansive industry has been documented by environmental monitoring and cleanup agencies [3, 9].

This study introduces a novel computational method for identifying manufactured gas production and storage sites (MGPs). Recently, geographers, historians, and other social scientists have begun to develop computational approaches to extract spatial data from primary historical primary sources including city directories [10], business registries [11], and maps [12]. Identifying and georeferencing historical MGP infrastructure at scale is important, for several reasons. First, while a handful of historical studies provide general accounts of the industry’s origins, evolution, and demise [4, 5, 13, 14], we are aware of no academic studies that locate manufactured gas production and storage infrastructure spatially at urban or regional scales. Doing so thus can offer basic insights into the industrial geography of America’s first gas industry and inform understanding of the spatial and temporal dynamics of energy transitions more generally [15–17]. Second, paired with historical sociodemographic data from the U.S. Census, urban-scale inventories of MGP infrastructure from the late 19th and early 20th centuries allow systemic investigation of environmental inequality during an era of rising racial and ethnic segregation [18–20]. With such data sets, social scientists can begin to study systematically the historical role of environmental threats, such as those posed by MGPs, in influencing the racial and ethnic stratification of American cities [21, 22].

A third, related but more pressing, reason is that MGPs produced enormous volumes of highly toxic and ecologically persistent wastes–principally including complex mixtures of coal tar and related petroleum hydrocarbons and a cocktail of heavy metals including cadmium, arsenic and mercury [23–25], though specific chemical signatures vary [26]. Unregulated by government agencies at the time, these wastes presented significant health hazards to plant workers and nearby residents [4]. Impacts were greater at central gasworks, but gas storage facilities can also be expected to harbor significant contaminants as well [3]. In addition, because federal legislation regulating hazardous industrial waste did not exist in the US until several decades after the industry’s demise, legacy wastes likely remain relatively sequestered at many of these legacy sites. Indeed, MGPs are routinely discovered by accident—perhaps as often as once a month across the US [3]—including the 1980 discovery in Stroudsburg, PA, of the first site to be listed under the EPA’s Superfund program [27]. MGPs are easily confirmed when excavated, either through their telltale infrastructure remnants or by the presence of signature chemical products and byproducts [3, 28]. However, the absence of efficient and systematic MGP identification methods means that state agencies and municipal governments lack the means to identify them easily prior to project implementation. Thus, the method we introduce below holds significant policy relevance in addition to creating opportunities for basic geographical and social science research.

## Overcoming data challenges to identifying historical MPG sites

Two previous efforts to identify and locate historical MGP sites at national and regional scales have faced significant data challenges. An EPA study published in 1985 [29] relied on Brown’s Directory of American Gas Companies [2] and estimated total U.S. sites to number about 1,500. However, because Brown’s identifies gas producers only by the cities in which gas companies were registered at time of publication and contains no street address information for production, storage, or distribution sites, the study underestimates its site count and fails to geolocate MPG sites (*Brown’s Directory* lists mailing addresses for gas company headquarters, but these offices were rarely located at production facilities). As noted above, remediation experts now understand this initial report to be a significant underestimation of the total number of extant MGP infrastructure scattered across US cities.

A second EPA study focusing on MGP sites in the Pacific Northwest [30] employed company information from Brown’s alongside manual inspection of historic Sanborn fire insurance maps. Pairing directory data with historic map documents, researchers geolocated thirty-five MGPs across EPA Region 10 (Alaska, Idaho, Oregon, and Washington), several of which were shown to present possible hazards to human health. Even so, manual map inspection is intensely time consuming, and pre-selecting searches from Brown’s directory listings misses secondary MGPs and storage and distribution sites. This limitation is underscored by the fact that in no case did the EPA Region 10 study identify more than one MGP site in any single urban area—even though historical accounts and reclamation data show that most cities were dotted with multiple sites hosting gas production and distribution infrastructure [1, 3].

**Fig 1:**
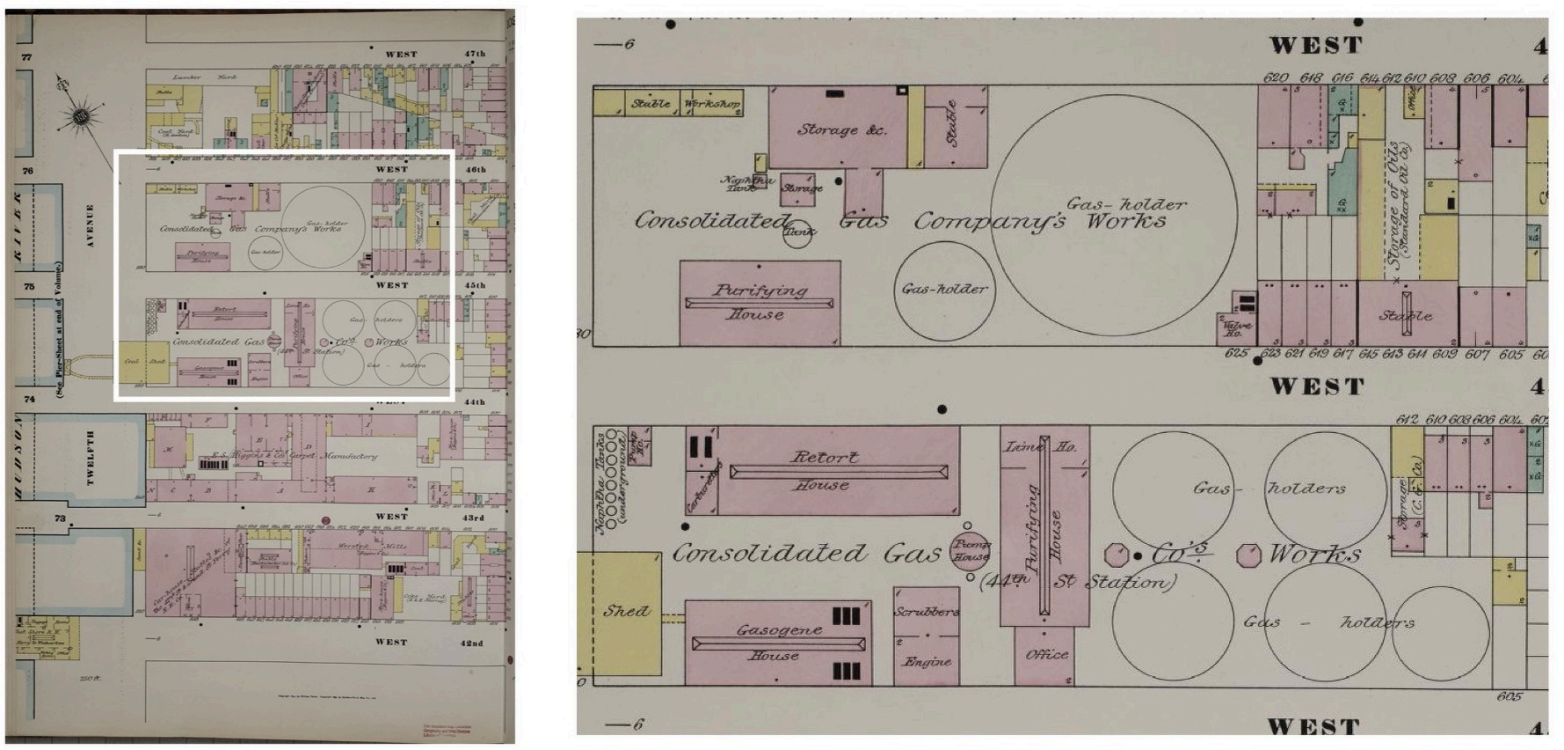
Circular gasometer structures as they appear in Sanborn fire insurance maps. Fig source: Sanborn Map Company.

We have developed a computational approach that significantly overcomes the data challenges hampering prior studies, allowing us to geolocate MGP infrastructure and identify smaller MGPs and secondary gas sites missed by the Region 10 approach. Our approach capitalizes on a distinct characteristic common to virtually all historic MGP sites: the presence of large, often multi-storied cylindrical structures used to store manufactured gas at central production plants and to maintain gas line pressure in outlying districts. On Sanborn maps, these cylindrical structures appear as open circles, often labeled as “gas holders,” “gasometers,” or “gas holding tanks” (see Fig 1). As circular gasometer structures were a necessary feature of all sites that produced or stored any quantity of manufactured gas, identifying gasometers is sufficient to locate above-ground manufactured gas infrastructure across the full range of production and distribution sites [3].

The Sanborn corpus is particularly well suited to MGP detection. Sanborn Co. published maps regularly for some 12,000 cities and towns beginning in the late 19th century through the 1960s, corresponding with the growth, height, and decline of the manufactured gas industry [3]. The spatial distribution of MGPs also matches the urban focus of the Sanborn map company: generally, MGPs were an urban phenomenon, with gas service scaling outward from more densely populated urban centers [1]. It is unlikely that a city large enough to support a manufactured gas industry would not have been regularly visited by Sanborn cartographers, who would have every reason to record the large, centralized storage tanks brimming with flammable gas.

Taking advantage of the circular architecture of MGP gasholders, we developed the Detect_MGP pipeline (https://github.com/TollefsonJ/Detect_MGP), which identifies circles on Sanborn map pages and selects those that correspond to MGP structures. It does so by using a Hough Transform to pass candidate circular regions to an ensemble machine learning model trained to identify the circular gasometer structures that signify MGPs and district gasometers. Our ensemble model, composed of five convolutional neural networks (CNN) and five multilayer perceptron models (MLP), autonomously extracts features associated with MGPs to distinguish between circular gasometers and other circular infrastructure such as water towers, oil tanks, and church domes. Sanborn atlases are particularly amenable to automated data extraction because they use relatively uniform scales, colors, labeling, and symbology across publication years and geography, and the tools we employ are well-tested: Hough transforms combined with deep learning methods have been shown to successfully detect and classify circular structures in other fields including biology, automation and robotics [31–33].

## MGP candidate extraction using the Hough transform

Machine learning applications rely on a consistent unit of analysis to produce meaningful results. Accordingly, this step of our analysis pipeline is designed to disaggregate complex map scans, which include a wide variety of building types, labels, text, and other information, into a collection of individual building-level images, allowing us to feed a consistent set of discretely coded building-level data into our ensemble machine learning classifier. We target circular infrastructure, specifically, because circular gasometer structures represent the most consistent and characteristic map item associated with MGP infrastructure. All MGPs contain at least one circular gasometer, and it is easy to extract circles from the overall “noise” of Sanborn map images.

The first step of our pipeline thus consists of extracting circular structures from Sanborn map images using the generalized Hough transform [34], using OpenCV’s (http://opencv.org) implementation of the transform, Hough.Circles. The Hough transform functions by constructing an N-dimensional accumulator array where N is defined by the number of unknown parameters. Circles are defined according to the equation below:

$${\text{r}}^{2}\;=\;{\left(\text{x}-\text{a}\right)}^{2}+{\left(\text{y}-\text{b}\right)}^{2}$$

Circles are represented by a 3-dimensional array corresponding to circle radius [r] and center location (a, b). Hough.Circles functions by defining a correspondence between a set of points in an image to a user-defined parameter space. For each set of points associated with a candidate circle, Hough.Circles collects votes in an accumulator array for parameters associated with a given candidate circle. Parameter values for radius (r) and location (a, b) corresponding to local maxima in the accumulator array are returned as identified circular extractions [35]. Hough transforms for identifying circular regions are relatively computationally expensive compared to the 2-dimensions that represent lines segments. Under the OpenCV implementation of a Hough Circle Transform, user-set parameters include the range of circle radii, the minimum distance between circle centers, and the accumulator threshold. Circle radii, distance, and accumulator threshold are set to minimize the number of false-positive identifications while ensuring that relevant candidate circles are not missed.

## Convolutional neural networks

To classify potential MGP structures extracted from map images by the Hough.Circles function outlined above, we draw upon an ensemble machine learning model that combines five convolutional neural networks with five multilayer perceptrons. Convolutional neural networks (CNN) are a type of feed-forward artificial neural network that has found broad application in environmental research and data processing [36–38] due in part to their capacity to extract an extremely large number of training features. CNNs are particularly suited to image classification tasks compared to other neural network models. While multilayer perceptron models analyze image data as an unspooled string of pixels values, CNNs preserve the spatial nature of training data by treating images as 2-dimensional objects. For this reason, CNN models are also translation invariant, and are better equipped to analyze image data that is skewed or otherwise imperfect.

CNN models distinguish among candidate images by identifying unique features associated with target image data. Feature sets are defined by iteratively constructing higher-order image features out of patterns recognized in lower-order features. Images are segmented into N x N kernels; each N x N kernel is simultaneously attached to F nodes representing F distinct features. To ensure translation invariance, the weights for each F feature for each N x N kernel are kept the same; CNNs learn by iteratively reducing the number of F nodes trained on F features for each image N x N kernel simultaneously. Many CNN architectures also introduce pooling layers between successive convolution iterations, which down-sample input feature maps by selecting the maximum values in a selected region [39] to maximize computational efficiency. A max-pooling function Yi = P(Xi), applied to channel i, is defined as:

$${\text{Y}}_{\text{i}}\left(\text{v}\right)\;=\;\text{Max}\left({\text{X}}_{\text{i}}\left(\overset{-}{\text{v}}\right):\overset{-}{\text{v}}\;\text{in}\;\text{neighbourhood}\;\text{of}\;\text{v}\right)$$

CNNs commonly output results to one or more fully connected layers, which contain all feature nodes from previous networks and perform a final series of operations to identify image features and return a designated classification or identification output. Many networks additionally include one or more dropout layers, which randomly remove network parameters to prevent overfitting.

## Multilayer perceptron

MLP models are arguably less specialized than CNN models. Used in a wide variety of machine learning applications due to their relative simplicity and low computational cost, MLP models consist of multiple hidden layers of interconnected neurons. Training consists of iteratively adjusting the weights associated with neuron connections through a process in which identification errors are backpropagated through neuron connections and weights are adjusted to minimize classification errors. Including MLP predictions in our ensemble learning approach produced a small increase in the efficacy of the full model while adding minimal computational overhead.

## MGP detection methods

**Fig 2:**
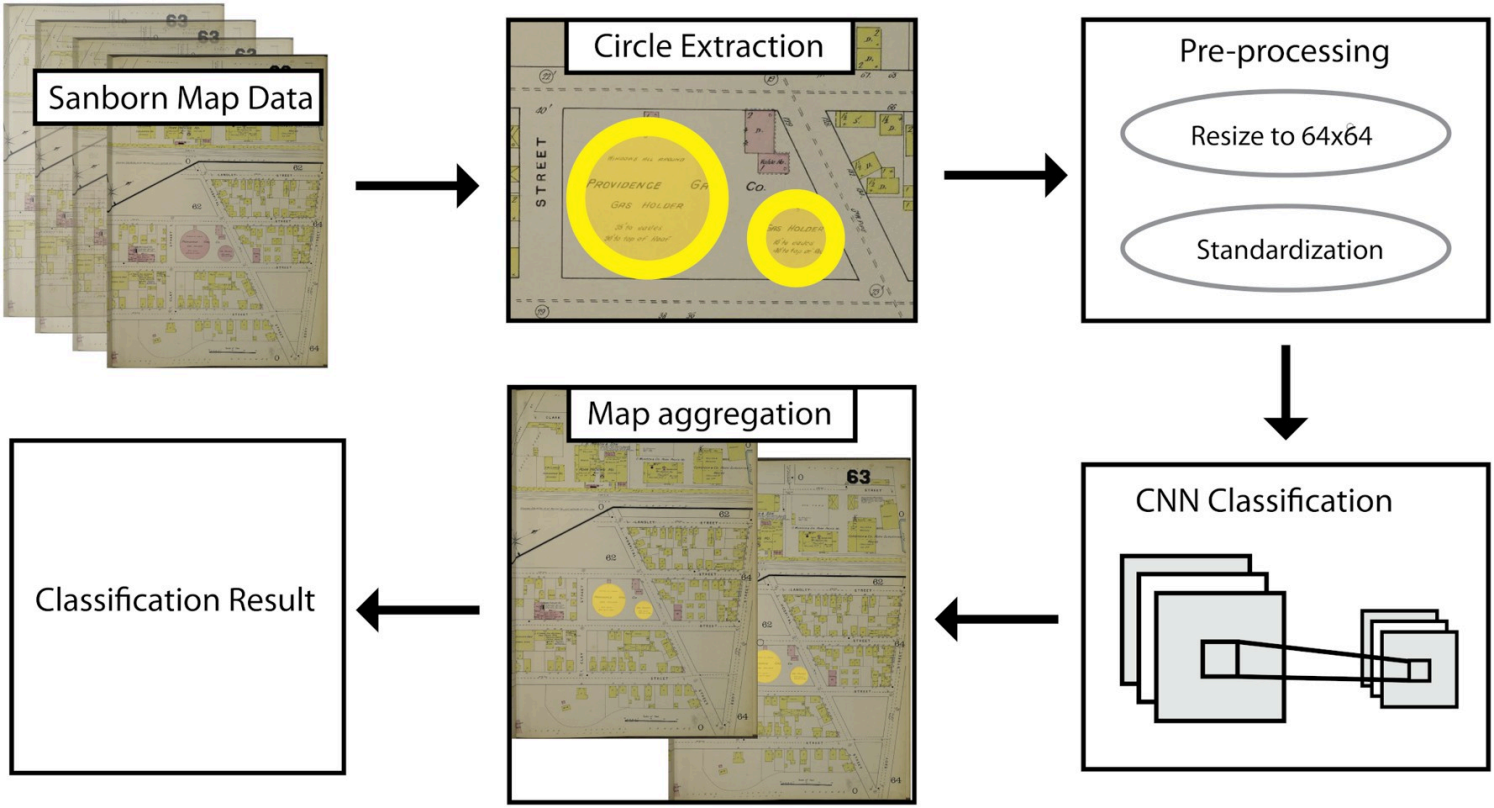
MGP identification workflow. Fig source: Tollefson.

Fig 2 displays the overall workflow, which consists of the following five steps: (1) Acquiring digital Sanborn map scans in bulk using the Library of Congress download API; (2) Extracting circular regions of interest of a given radius by applying the Hough.Circles algorithm to Sanborn map images; (3) Resizing circular regions to 64 x 64 pixels and standardizing pixel values; (4) Classifying extracted circular regions as MGP or non-MGP infrastructure using an ensemble machine learning classifier; and (5) Returning full map pages for final visual inspection. The following sections describe each of these steps.

### Data acquisition

Digitized Sanborn atlases are composed of multiple map volumes each numbering several hundred pages and stored as individual JPG files. Each map page represents a few square blocks of city space. The Library of Congress (LOC) Sanborn Maps Collection hosts high quality, full color map scans used for this study. The collection holds nearly 35,000 volumes totaling hundreds of thousands of map pages. Map volumes are publicly available for download as a structured folder system by submitting an API request to the Library of Congress database. Scans are delivered at a resolution of 1462 x 2067 pixels, which contains enough detail to identify descriptive labels while minimizing the costs to store and process image data. We processed 16,393 individual map pages through the Detect_MGP pipeline. We selected map data from Chicago, San Francisco, Portland, New Orleans, the New York City area, and the state of Rhode Island. Map data were selected to cover a range of publication years and geographic locations to account for variation in map style (see Table 1). While Sanborn maps are remarkably consistent, we noted minor variations in coloration and font between the earliest and latest map volumes we examined. The distribution of circular map features also varies slightly by region: San Francisco, for instance, includes a relatively high density of water towers and cisterns, while many smaller Rhode Island towns include circular name labels similar in size to large gasometer structures. Aggregating map images from a diverse spatial and temporal sample allows us to input a wide array of circular map features into our CNN algorithm, increasing the overall generalizability of the Detect_MGP pipeline.

**Table 1:** Map volumes from sampled cities and regions.

Region	Years
Chicago	1901, 1905, 1906, 1908, 1909, 1910, 1911, 1912, 1913, 1914, 1916, 1917, 1918, 1919, 1920, 1946, 1949, 1950, 1951
New Orleans	1885, 1887, 1893, 1895, 1896, 1908, 1909, 1937, 1940, 1950, 1951
New York City area	1886, 1887, 1888, 1893, 1895, 1890, 1891, 1892, 1893, 1894, 1895, 1896, 1897, 1898, 1899, 1898, 1901, 1902, 1903
Portland	1889, 1901, 1905, 1908, 1909, 1950
Rhode Island (all cities)	1884, 1885, 1886, 1887, 1889, 1890, 1891, 1892, 1894, 1896, 1897, 1898, 1899, 1900, 1902, 1903, 1907, 1909, 1910, 1911, 1912, 1920, 1921, 1922, 1923, 1925, 1928, 1933, 1934, 1935, 1940, 1941, 1944, 1945, 1946, 1947, 1949, 1950, 1951, 1953, 1955, 1956, 1958
San Francisco	1886, 1887, 1889, 1893, 1899, 1900, 1913, 1914, 1915, 1948, 1949, 1950

Note: Publication dates represent years in which at least one map volume was published in a given region. New York City area truncated at 1903.

### MGP candidate detection

As noted above, we rely on the Hough.Circles circle identification tool to extract building-level information from complex Sanborn map images in order to provide our machine learning tools a consistent corpus of classification data. In addition, circle identification removes map images that do not contain regions that may be associated with circular gasometers, and thus serves as a strong initial filter to narrow the range of possible MGP locations. The Hough.Circles step extracted 6682 circular structures from the 16,393 map pages sized 1462 x 2067 pixels, using a range of 15 to 130 pixels as the target circle radius. Circle radii and Hough.Circles parameters were selected to ensure a minimal rate of false negatives; as a result, the 6682 circles identified include a relatively high number of non-circular patterns, including semicircular street segments and blocks with high density of building outlines. The 6682 circles represent 3894 map pages across 278 map volumes, covering the full range of publication years from 1884 to 1958. We programmed the Hough.Circles function to return a square image 20% wider than circle radii to preserve valuable information from the map regions immediately surrounding candidate circles.

### Pre-processing

Extracted regions are resized to 64 x 64 pixels, which are represented as a three-dimensional array of pixel values representing red, green, and blue color fields. Regions are then converted to grayscale, reducing each image to a one-dimensional array. While the CNN approach to classification allows for full-color image analysis, our results in this case do not suggest that RGB images produce more accurate classification results. For the CNN component of the hybrid model, pixel values are then scaled to fall between 0 and 1 by dividing each pixel value by 255, as convolutional neural networks perform better using scaled and normalized input data [40]. The MLP component transforms input data using Scikit-Learn’s StandardScaler function, which scales input data by subtracting the mean pixel value and dividing by unit variance.

### Hybrid model

**Fig 3:**
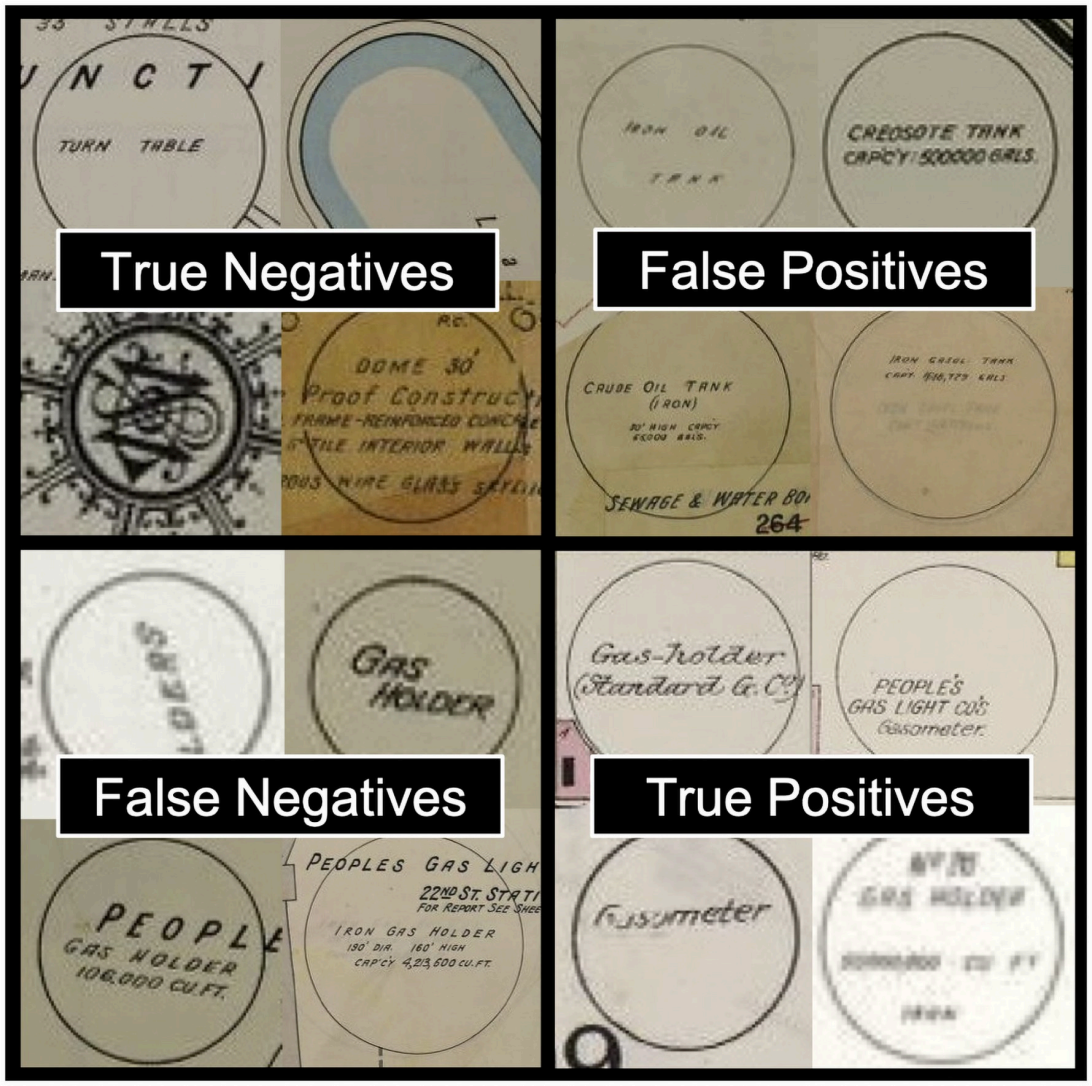
Examples of positive and negative MGP classification categories. Fig source: Tollefson, using map images from Sanborn Map Company.

We used the 6,682 MGP candidates extracted by the Hough.Circles function to train and test a hybrid CNN-MLP model in order to distinguish between MGP and non-MGP infrastructure, using the Keras machine learning library based on a TensorFlow CNN backend and the Scikit-Learn implementation of an MLP classifier. We coded each of the identified candidate circles as a binary classification (MPG/non-MGP), resulting in 335 “positive” cases and 6,384 “negative” cases (see Fig 3).

We used a K-fold cross-validation approach to train and test our hybrid model. K-fold cross-validation is a standard tool to control for variation in model training results caused by the random assignment of input data to training and testing sets. It functions by splitting input data into K bins; K separate models are trained on K-1 bins, with the final bin withheld to test model training results. A five-fold cross-validation approach produces five separate model test results, each of which is tested on 20% (N = 1,336) of the total training data. For CNN model training, the four folds selected for each model training iteration, representing 80% of the dataset in total, were further split into training and validation samples (85%, N = 4,544 and 15%, N = 802, respectively). Validation data are used to iteratively assess CNN model fit across all 80 training epochs–an approach that provides a visual measure of possible overfitting.

CNN architecture is outlined in Table 2 below. Our final CNN model consists of an input layer, four convolutional layers for feature extraction, three pooling layers to downsample parameters, and three fully connected layers; we also include a dropout layer with a ratio of 50% to reduce overfitting. Batch size was set to 64, and “Relu” activation functions were used for each of the convolution layers. Hyper-parameters—including the selection of model layers, activations functions, and kernel sizes—were selected from a series of candidate models using best-practice approaches to model design. The “Adamax” algorithm, a variant of the more common “Adam” algorithm, was selected as the model optimizer, as it exhibited slightly stronger performance. We tested multiple CNN architectures before arriving at the model outlined below. These variants included up to three additional pairs of convolutional and pooling layers beyond those listed below; batch sizes of 32, 64, 128, and 248; and 3-dimensional full-color image inputs, as well as the 1-dimensional grayscale image set used here.

**Table 2:** CNN layer architecture.

Layer	Output shape	Param. #
2D Convolution	[64, 64, 16]	448
2D Convolution	[62, 62, 16]	2,320
Max Pooling	[31, 31, 16]	
2D Convolution	[29, 29, 32]	4,640
Max Pooling	[14, 14, 32]	
2D Convolution	[12, 12, 64]	18,496
Max Pooling	[6, 6, 64]	
Flatten	[2304]	
Dense	[1032]	2,378,760
Dropout	[1032]	
Dense	[2]	2,066
Total parameters		2,406,730

MLP hyperparameters were selected using a “grid search” approach, which tests multiple combinations of a user-set parameter space. MLP hyperparameters are listed in Table 3, with final selections indicated with an asterisk. Multiple algorithms are available to guide weight adjustment; we selected the LBFGS algorithm, as it is especially suited to smaller datasets.

**Table 3:** MLP hyperparameters.

Parameter	Tests
Hidden layer sizes	(100, 50)*
	(100,)
	(3, 3, 3)
Alpha	0.00001*
	0.0001
	.01
	0.1
	0.5
Learning rate	Constant*
	Adaptive

* Selected parameter.

#### Class imbalance

As noted above, our training data exhibit strong class imbalance. Imbalanced training data presents a particular challenge for data classification algorithms: Applying standard accuracy metrics to unbalanced data will produce a strong bias for the majority case. We applied two separate approaches to controlling for class imbalance for the CNN and MLP components of our hybrid model. To account for class imbalance in the CNN model training, we applied a simple weighting function—included as a user-set hyperparameter in the Keras CNN implementation—which effectively increases the cost of each inaccurate classification of the minority case. In the model outlined below, positive cases were weighted at 19.2 per negative case unit to account for the imbalance in model training data. For the MLP model, we relied on a synthetic oversampling technique implemented with the “Imbalanced-learn” (or “Imblearn”) package (http://www.imbalanced-learn.org). Imblearn generates new minority case data according to a number of different user-set algorithms. We tested 25%, 50%, and 100% oversampling ratios using the popular “synthetic minority over-sampling technique” (or “SMOTE”) package; this approach reduced the ratio of minority to majority cases from the original 1:20 ratio to 1.25:20, 1.5:20, and 2:20, respectively. Following the minority oversampling step, training data were balanced at a 1:1 ratio by randomly undersampling majority-class cases. Like MLP classifiers, Imblearn oversampling tools depend on one-dimensional data inputs; we did not apply Imblearn oversampling tools to CNN input data, as doing so would require “throwing out” the two-dimension spatial relationship between image pixels.

#### Ensemble learning

We draw on ensemble learning strategies to increase prediction accuracy. Ensemble approaches average the predictions from multiple models trained and tested on the same dataset. We produced 5 separately trained CNN and MLP models constructed using the architecture outlined in Tables 2 and 3, respectively. We integrate predictions produced by the CNN and MLP models through two steps. First, we produce separate mean prediction arrays for the CNN and MLP models. Then, each mean prediction array is scaled according to its respective optimal prediction thresholds, calculated to maximize the difference between true positive and false positive rates. We use the average of these two-scaled predictions to produce a final prediction output, and calculate a second optimal prediction threshold in the same manner (see equation below). See the following section for further discussion on selecting an optimal prediction threshold.


$$\text{P}\left(\text{final}\right)\;=\;\frac{\frac{\text{T}\left(\text{MLP}\right)*\text{P}\left(\text{CNN}\right)}{\text{P}\left(\text{MLP}\right)}+\frac{\text{T}\left(\text{CNN}\right)*\text{P}\left(\text{MLP}\right)}{\text{P}\left(\text{CNN}\right)}}{2}$$

#### Model training results

The ensemble model described above was trained using free GPU runtime hosted on a Google Colab environment. Each CNN classifier required 80 seconds to train, while each MLP classifier required an additional 10 seconds; 25 ensemble models [5 models * 5 folds] were trained in total, a 38-minute total runtime. Results are evaluated below.

Two metrics track the efficacy of the CNN model through its 80 training iterations. Accuracy is defined as the percentage of successful classifications, weighted to account for the imbalance between positive and negative training cases; Keras accuracy calculations use 0.5 probability as the cutoff for binary predictions during model training. See equation below, where ${\hat{\text{y}}}_{\text{i}}$ represents the predicted classification and y_i_ the true classification for case *i*:

$$\left\{\begin{array}{cc} {\hat{\text{y}}}_{\text{i}}\;=\;1, & \text{if}\;\text{P}\left({\text{y}}_{\text{i}}\;=\;1\right)\geq 0.5\\ {\hat{\text{y}}}_{\text{i}}\;=\;0, & \text{if}\;\text{P}\left({\text{y}}_{\text{i}}\;=\;1\right)<0.5 \end{array}\right.$$

We also use a binary cross-entropy loss function to return a continuous calculation of model error throughout the training period. Each of the five CNN models produced approximately 90% accuracy, with approximately 90% accuracy observed on the validation dataset as well. Loss calculations for each sub-model were within several points of 15% on the training set and 15% on the validation set, respectively. Despite some irregularity caused by the relatively low frequency of positive training cases, training and validation datasets returned comparable accuracy and loss functions over the 80 training epochs, indicating that our trained model did not experience strong overfitting.

**Fig 4:**
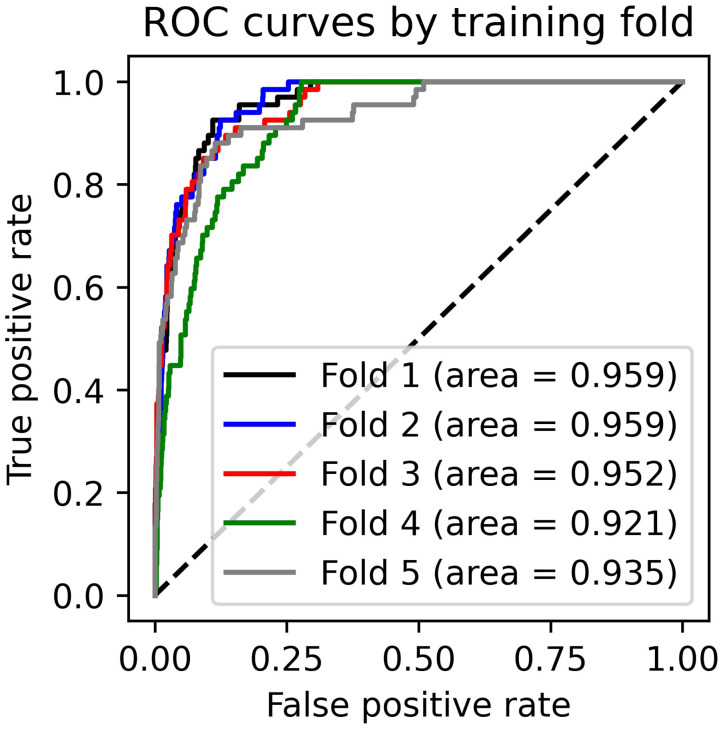
ROC curves for all folds and all model iterations, hybrid CNN-MLP model. Dotted line displays expected TRP-FPR curve for a fully random classifier. Fig source: Tollefson.

Fig 4, below, presents ROC curves for test-data predictions for all five folds for the hybrid CNN-MLP model. ROC curves display the relationship between the rate of true positive identifications compared to false positive identifications across the full range of classification thresholds. The “area” value presents the area under the ROC curve for each testing fold. Area-under-curve (AUC) values therefore provide a useful metric to compare the effectiveness of different predictive models; the hybrid CNN-MLP model was selected because it slightly out-performed the individual CNN and MLP components as measured on the AUC metric, and produced less variable results across training folds (AUC for the CNN-MLP model ranged from 0.92 to 0.96; 0.85 to 0.94 for the MLP model alone; and 0.89 to 0.94 for the CNN model alone). ROC curves have the additional benefit of providing a simple tool to select an optimal threshold value for predicting class labels: The optimal threshold value is the one that produces a true positive—false positive distribution at the upper left corner of the ROC curve, representing the best-performing tradeoff between model accuracy and precision.

### Full-size map images

As a final step, circular regions were aggregated up to full-size map images for final visual confirmation. As many Sanborn map pages contain multiple MGP and non-MGP circular regions, the total number of map pages that require visual inspection is less than the total number of circles that the Detect_MGP pipeline extracted and classified. The 1,336 test samples analyzed by our Detect_MGP pipeline represent an average of 1,075 total map pages across the five test folds. Since false-negatives may be located on the same map pages as true positives–and because MGP infrastructure is often located near other industrial infrastructure that represent the bulk of the false-positive outputs–page-wise visual confirmation potentially allows us to recover MGP sites that were mis-classified. Across the five test folds, an average of three MGP sites per fold were recovered in this fashion, raising the average recall rate of the full model (the rate at which the model successfully identifies true-positives) from 84% to nearly 90%.

## Results and discussion

This study employs the Hough.Circles circle detection algorithm to identify circular map structures in historic Sanborn insurance maps that represent potential MGP sites, and passes them through a hybrid CNN-MLP model to distinguish between candidate circular regions in order to separate MGPs from non-MGP circular infrastructure. Final results from each pipeline step, using data associated with Fold 1 of the test dataset, are summarized in Table 4, which outlines the stepwise reduction in candidate map pages and candidate circles for each stage in the Detect_MGP pipeline. In summary, the Detect_MGP approach decreases the time required to assess Sanborn map pages by 94%: For a corpus of 100,000 map documents, assuming that visual inspection requires an average of five second per page, Detect_MGP reduces the total coding time from an estimated 138 hours to just under seven. The method allows for extraction of precise MGP location data on greatly increased geographic and temporal scales; paired with a final manual inspection to reduce false positives, the model achieves a recall rate of just under 90%.

**Table 4:** Step-wise reduction in candidate map pages.

Step	N-output	N-MGP	Pct. Positive
1. Data acquisition	3,278(m)	56(m)	1.7%
2. Circle detection	1,336(c)	68(c)	5%
3. Standardization	-	-	-
4. CNN + MLP model	206(c)	58(c)	28%
5. Full-size map images	166(m)	50(m)*	30%
Total reduction in candidate map regions: 94%			
Final recall rate: 90%			

(m) N = number of full map pages.

(c) N = number of candidate circles

* No MGP data loss in this step. Reduction in N-MGP from 58 to 50 due to aggregation of individual candidate circles to full map pages.

Because the hybrid CNN-MLP model was tested on a subset of the total number of map pages analyzed, we center the final results estimate on the mean output of the five tests of the CNN-MLP classifier (N = 1,336 regions), which represent one-fifth of the total number of candidate circles (N = 6,682) extracted from full set of 16,393 map pages. Steps 2–4 therefore present real-world results based on five-fold CNN-MLP testing data. To allow for meaningful comparison across all four steps of the Detect_MGP pipeline, Table 4 scales the number of input map pages according to the one-fifth testing fold presented in steps 2–4.

Because the Sanborn Co. mapped many MGPs multiple times across sequential map series, our estimated 90% recall rate is likely a minimum, as some of the relatively small number of MGPs overlooked by the Detect_MGP pipeline may be located by identifying them in Sanborn map images from different publication years. Results may be further improved by implementing optical character recognition (OCR) alongside a machine learning approach to MGP identification: Additional OCR analysis might provide a method to further filter CNN outputs or, if applied to map pages as a whole, may locate MGP-related map labels that fall outside of circular map regions.

The Detect_MGP method is reliant on the geographic scope and historical accuracy of Sanborn maps and the regularity of map production, which introduces certain limitations. First, the Sanborn Company was focused on assessing urban fire risk, so their mapping operation at times failed to capture non-urban or less densely developed urban areas [41]. However, because the manufactured gas infrastructure predominantly served an urban and industrial customer base [3], we expect existing Sanborn maps to include a large majority of sites that physically existed when Sanborn cartographers surveyed those places. A related limitation involves the Sanborn Map Company’s irregular publication schedule and the limited availability, for some cities and years, of a city’s complete map series [41].

Because our approach relies solely on Sanborn documents, it may not capture MPG sites that opened and closed between map publication years. Concern here is mitigated by the fact that Sanborn maps capture building footprints whether or not those buildings were in use at the time of publication. This means that MGP sites might be identified long after closure so long as the physical gasometer or gas holder structures remained standing. Despite these limitations, Sanborn maps remain the most complete and accurate source of building-level information for the regions and time-period in which the US manufactured gas industry was operating.

Identifying sites through an inductive analysis of the full Sanborn corpus for a given city or region also allows us to locate former MGPs that may be missed by approaches that target specific sites based on directory data or other information. The EPA Region 10 study, noted at the beginning of this article, identified only a single MGP site in any given city, locating 4 in Idaho, 10 in Oregon, and 14 in Washington [30]. When we applied the Detect_MGP pipeline to Sanborn maps for Washington, totaling 9,969 pages, we located 23 separate MGP sites in 15 different cities–a significant improvement over the prior study. With Detect_MGP, a single researcher can render such improvements in a few hours.

Inductive site investigation enables researchers to systematize the discovery of environmental hazards that faded from the urban landscape prior to rigorous site investigation requirements. Here, the timing of the manufactured gas industry’s decline carried measurable consequences: Using Sanborn map data to locate former gas plants in the Providence, RI area and using additional historical records to investigate prior and current uses for each site, identified 19 MGPs, just three of which are listed in state or federal monitoring databases [7]. Beyond locating specific lost gasworks, our tool also provides historical geographers and other researchers the ability to centralize the process of historic site identification, enabling historical-spatial research at any scale and in any location–regardless of the progress of local municipalities or property owners to identify the previous uses of individual parcels.

We hope that the tools described in this paper will be useful to scholars interested in historical urban and industrial geography as well as land use researchers tasked with identifying potential environmental and health hazards in American cities [6]. Similar approaches may also be applied to identify other prominent map items, including former sites of industrial or manufacturing use, many of which include specific circular map regions (including diesel, oil, tar, or chemical tanks), or are rendered in distinct line types or color schemes. Non-circular infrastructure may also be identified and classified by altering the Hough transform to search for features that match the item of interest, or by applying a mixed approach using tools to identify geometric features paired with OCR analysis. As their cartographic characteristics remain largely unchanged over time and across map geographies, Sanborn maps are promising data sources for longitudinal and comparative feature classification for a wide range of urban infrastructure.
